# Supplementary material for: The Small RNA Universe of Capitella teleta
Source: Front Mol Biosci. 2022 Feb 25;9:802814. doi: 10.3389/fmolb.2022.802814 (PMC8915122; doi:10.3389/fmolb.2022.802814)
Supplement: Supplementary file 1 [file DataSheet1.ZIP › Supplement/known/CAPTEscaffold_174_10475.pdf]

Provisional ID	:	CAPTEscaffold_174_10475
Score total	:	76628.6
Score for star read(s)	:	3.9
Score for read counts	:	76618.7
Score for mfe	:	1.4
Score for randfold	:	1.6
Score for cons. seed	:	3
Total read count	:	150296
Mature read count	:	149623
Loop read count	:	176
Star read count	:	497

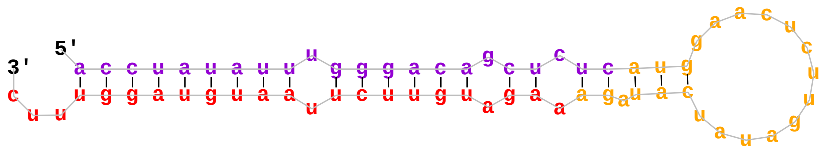

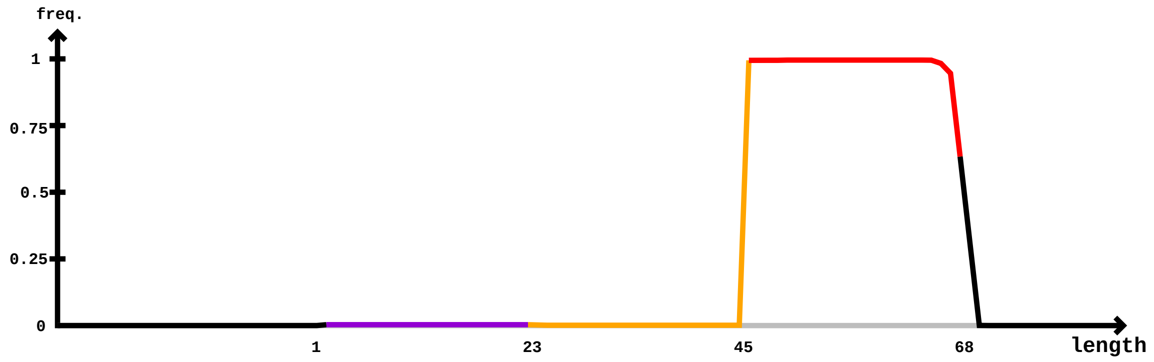


```
                           Star                                         Mature
5'- ugagcccucgcggucauaucacaagaaccuauauuugggacagcucucauggaacucuugauaucauagaaagauguucuuaauguagguuucuugugcugaccuuuau  -3'  obs
    ugagcccucgcggucauaucacaagaaccuauauuugggacagcucucauggaacucuugauaucauagaaagauguucuuaauguagguuucuugugcugaccuuuau       exp
    ugagcccucgcggucauaucacaagaaccuauauuugggacagcucucauggaacucuugauaucauagaaagauguucuuaauguagguuucuugugcugaccuuuau       known
    ...((....))(((((...((((((((((((((((.((((((.((.(((((.............))).)).)).)))))).)))))))).)))))))).))))).....  reads    mm      sample

    ..........................accuauauuugggacagc.................................................................  2        0       seq
    ..........................accuauauuugAgacagcucu..............................................................  1        1       seq
    ..........................Gccuauauuugggacagcucu..............................................................  1        1       seq
    ..........................accuauauuugggacagcucu..............................................................  17       0       seq
    ..........................acAuauauuugggacagcucu..............................................................  1        1       seq
    ..........................accuauauuuAggacagcucuc.............................................................  4        1       seq
    ..........................Gccuauauuugggacagcucuc.............................................................  4        1       seq
    ..........................accuauauuugggacagcucuc.............................................................  457      0       seq
    ..........................accuauauuugggaGagcucuc.............................................................  1        1       seq
    ..........................accuauauuugggacagcucNc.............................................................  1        1       seq
    ..........................accuauauuAgggacagcucuc.............................................................  1        1       seq
    ..........................accuauauuugAgacagcucuc.............................................................  2        1       seq
    ..........................accuauauuugggacagcucucU............................................................  5        1       seq
    ................................................auggaacucuugauauca...........................................  1        0       seq
    ................................................auggaacucuugauaucauag........................................  1        0       seq
    ................................................auggaacucuugauaucauaga.......................................  173      0       seq
    ................................................auggaacucuuAauaucauaga.......................................  1        1       seq
    ....................................................................Aaaagauguucuuaauguagguuuc................  2        1       seq
    .....................................................................Caagauguucuuaauguagguu..................  1        1       seq
    .....................................................................Caagauguucuuaauguagguuu.................  3        1       seq
    .....................................................................Caagauguucuuaauguagguuuc................  4        1       seq
    ......................................................................aagaugAucuuaauguagg....................  1        1       seq
    ......................................................................aagauguucuuaauguagg....................  56       0       seq
    ......................................................................aagauguucuuaauguaCgu...................  1        1       seq
    ......................................................................aagauguucuuUauguaggu...................  17       1       seq
    ......................................................................aagauguucuAaauguaggu...................  1        1       seq
    ......................................................................aagaGguucuuaauguaggu...................  1        1       seq
    ......................................................................aagauguucuCaauguaggu...................  6        1       seq
    ......................................................................aagaugAucuuaauguaggu...................  1        1       seq
    ......................................................................aagauguucuuaaugAaggu...................  1        1       seq
    ......................................................................aagauguucuuaauguaggu...................  1656     0       seq
```

```
                              Star                                                   Mature
ugagcccucgcggucauaucacaagaaccuauauuugggacagcucucauggaacucuugauaucauagaaagauguucuuaauguagguuucuugugcugaccuuuau
......................................................................Uagauguucuuaauguaggu...................   1       1   seq
......................................................................aNgauguucuuaauguaggu...................   1       1   seq
......................................................................aagauguucuuaauguaggA...................   140     1   seq
......................................................................aagauguuGuuaauguaggu...................   1       1   seq
......................................................................aagauguucAuaauguagguu..................   2       1   seq
......................................................................aagauguAcuuaauguagguu..................   1       1   seq
......................................................................aagauguucuuaaugGagguu..................   1       1   seq
......................................................................aagauguuUuuaauguagguu..................   1       1   seq
......................................................................Uagauguucuuaauguagguu..................   1       1   seq
......................................................................aaAauguucuuaauguagguu..................   1       1   seq
......................................................................aagauguucuAaauguagguu..................   6       1   seq
......................................................................aagaugAucuuaauguagguu..................   6       1   seq
......................................................................aagaAguucuuaauguagguu..................   1       1   seq
......................................................................aagauguucuuaaugAagguu..................   1       1   seq
......................................................................aagauguucuuaauguagguu..................   5436    0   seq
......................................................................aagauguuAuuaauguagguu..................   2       1   seq
......................................................................aagauguucuuaauguagUuu..................   1       1   seq
......................................................................aagauguucuuaaugCagguu..................   1       1   seq
......................................................................aagauguGcuuaauguagguu..................   1       1   seq
......................................................................aUgauguucuuaauguagguu..................   2       1   seq
......................................................................aagauguucuuaGuguagguu..................   2       1   seq
......................................................................aagUuguucuuaauguagguu..................   1       1   seq
......................................................................aagaugGucuuaauguagguu..................   3       1   seq
......................................................................aagGuguucuuaauguagguu..................   1       1   seq
......................................................................aagauguucCuaauguagguu..................   2       1   seq
......................................................................aagauguucuuaauguagguC..................   2       1   seq
......................................................................aagauguucuuaauguaUguu..................   1       1   seq
......................................................................aagauguucuuaauguaAguu..................   2       1   seq
......................................................................aagauguucuuaauguagguG..................   2       1   seq
......................................................................aagauguucuuaauguaggCu..................   38      1   seq
......................................................................aagauguucuuaauguaCguu..................   1       1   seq
......................................................................aagauguucuuUauguagguu..................   9       1   seq
......................................................................aNgauguucuuaauguagguu..................   1       1   seq
......................................................................aagauguCcuuaauguagguu..................   2       1   seq
......................................................................aagaCguucuuaauguagguu..................   1       1   seq
......................................................................aagaugCucuuaauguagguu..................   2       1   seq
......................................................................Gagauguucuuaauguagguu..................   2       1   seq
......................................................................aagaGguucuuaauguagguu..................   1       1   seq
......................................................................aagauguucuuaauguagguA..................   12      1   seq
......................................................................aagauguucuuaaAguagguu..................   1       1   seq
......................................................................aagauguucuCaauguagguu..................   8       1   seq
......................................................................aagaCguucuuaauguagguuu.................   4       1   seq
......................................................................aagauguucuuaauguaggAuu.................   17      1   seq
......................................................................aagauguucuuaauAuagguuu.................   6       1   seq
......................................................................aagauguucuuaauguagguuu.................   46373   0   seq
......................................................................aagauNuucuuaauguagguuu.................   1       1   seq
......................................................................aagauguucuuaauCuagguuu.................   3       1   seq
......................................................................aagauguuAuuaauguagguuu.................   10      1   seq
......................................................................Nagauguucuuaauguagguuu.................   16      1   seq
......................................................................aagauguucuuaauguagUuuu.................   2       1   seq
......................................................................aagauguucuuaauguaggGuu.................   12      1   seq
......................................................................aagauguucuuaGuguagguuu.................   10      1   seq
......................................................................aagauguucuuaauguaAguuu.................   10      1   seq
......................................................................aagauguucAuaauguagguuu.................   15      1   seq
......................................................................aagauguucuuaauguagguuG.................   27      1   seq
......................................................................aaAauguucuuaauguagguuu.................   2       1   seq
......................................................................aagauguucuuaUuguagguuu.................   7       1   seq
......................................................................aagauguucuuaCuguagguuu.................   1       1   seq
......................................................................aagauguucuuaauguagguuA.................   56      1   seq
......................................................................aGgauguucuuaauguagguuu.................   8       1   seq
......................................................................aagauguucuuaaugAagguuu.................   10      1   seq
......................................................................aagauguucCuaauguagguuu.................   4       1   seq
......................................................................aagauguucuGaauguagguuu.................   2       1   seq
......................................................................aagauguucuuaauguagguuC.................   11      1   seq
......................................................................aagauguucuuUauguagguuu.................   2       1   seq
......................................................................aagauguucuuaauguaggCuu.................   59      1   seq
......................................................................aagauguucuuaauUuagguuu.................   4       1   seq
......................................................................aagauguCcuuaauguagguuu.................   6       1   seq
......................................................................aagauguucuuaaugGagguuu.................   5       1   seq
......................................................................aagauguAcuuaauguagguuu.................   17      1   seq
```

Star Mature

```
ugagcccucgcggucauaucacaagaaccuauauuugggacagcucucauggaacucuugauaucauagaaagauguucuuaauguagguuucuugugcugaccuuuau
......................................................................aagauguucuuauguUgguuu.................	4	1	seq
......................................................................aagauAuucuuaauguagguu.................	9	1	seq
......................................................................aagUuguucuuaauguagguuu.................	5	1	seq
......................................................................aagauguucuuaauguaUguuu.................	5	1	seq
......................................................................aagauguuUuuaauguagguuu.................	10	1	seq
......................................................................aagauguucGuaauguagguuu.................	5	1	seq
......................................................................aagauguucuuaauguNgguuu.................	1	1	seq
......................................................................aagauguucuuaaugCagguuu.................	4	1	seq
......................................................................aagauguucuuGauguagguuu.................	6	1	seq
......................................................................aagauguuGuuaauguagguuu.................	5	1	seq
......................................................................aagauguucuuaauguagguAu.................	8	1	seq
......................................................................aagauguucuuaaAguagguuu.................	11	1	seq
......................................................................aagaGguucuuaauguagguuu.................	3	1	seq
......................................................................aagauguucuCaauguagguuu.................	16	1	seq
......................................................................aNgauguucuuaauguagguuu.................	1	1	seq
......................................................................aaUauguucuuaauguagguuu.................	4	1	seq
......................................................................aaCauguucuuaauguagguuu.................	1	1	seq
......................................................................Gagauguucuuaauguagguuu.................	14	1	seq
......................................................................aagauguucuuaauguGgguuu.................	8	1	seq
......................................................................aagaugCucuuaauguagguuu.................	8	1	seq
......................................................................aagauguucuuaauguagguGu.................	4	1	seq
......................................................................aagaugAucuuaauguagguuu.................	33	1	seq
......................................................................aagauguGcuuaauguagguuu.................	1	1	seq
......................................................................aagauUuucuuaauguagguuu.................	1	1	seq
......................................................................aagaugGucuuaauguagguuu.................	9	1	seq
......................................................................aagaAguucuuaauguagguuu.................	28	1	seq
......................................................................aagGuguucuuaauguagguuu.................	3	1	seq
......................................................................aagCuguucuuaauguagguuu.................	1	1	seq
......................................................................aagauguucuuaauguagAuuu.................	2	1	seq
......................................................................aagauguucuAaauguagguuu.................	18	1	seq
......................................................................aagauguucuuaauguagguCu.................	4	1	seq
......................................................................aUgauguucuuaauguagguuu.................	3	1	seq
......................................................................aagauguucuuaaCguagguuu.................	5	1	seq
......................................................................aagauguucuuaaGguagguuu.................	3	1	seq
......................................................................aagauguucuuUauguagguuuc................	9	1	seq
......................................................................aagauguucuuaauUuagguuuc................	5	1	seq
......................................................................aagauguucAuaauguagguuuc................	30	1	seq
......................................................................aagauguucuuaauguagguGuc................	6	1	seq
......................................................................aagauguucuuaaGguagguuuc................	3	1	seq
......................................................................aagauguucuuaauguagCuuuc................	3	1	seq
......................................................................Nagauguucuuaauguagguuuc................	21	1	seq
......................................................................aagauguucGuaauguagguuuc................	6	1	seq
......................................................................aGgauguucuuaauguagguuuc................	10	1	seq
......................................................................aagauguCcuuaauguagguuuc................	8	1	seq
......................................................................aagauguucuuaauguagguuuG................	6	1	seq
......................................................................aagauguucuuaauguagguuuA................	139	1	seq
......................................................................aagauguucuuaauguagguuCc................	5	1	seq
......................................................................aagauguucuuaauguagguuuc................	86036	0	seq
......................................................................aagaugGucuuaauguagguuuc................	12	1	seq
......................................................................aagauAuucuuaauguagguuuc................	12	1	seq
......................................................................aagauguucuuaauguaggGuuc................	12	1	seq
......................................................................aagauguucuuaauguUgguuuc................	5	1	seq
......................................................................aagauguucuuaauguagguuGc................	2	1	seq
......................................................................aaUauguucuuaauguagguuuc................	5	1	seq
......................................................................aagauguucuuaUuguagguuuc................	8	1	seq
......................................................................aNgauguucuuaauguagguuuc................	3	1	seq
......................................................................aagGuguucuuaauguagguuuc................	7	1	seq
......................................................................aaAauguucuuaauguagguuuc................	1	1	seq
......................................................................aagaugAucuuaauguagguuuc................	60	1	seq
......................................................................aagauguGcuuaauguagguuuc................	2	1	seq
......................................................................aagauguucuuaauAuagguuuc................	8	1	seq
......................................................................aaCauguucuuaauguagguuuc................	4	1	seq
......................................................................aagauguucuuaauguagUuuuc................	6	1	seq
......................................................................aagauguucuuaCuguagguuuc................	2	1	seq
......................................................................aagauguucuuaauguaUguuuc................	3	1	seq
......................................................................aagUuguucuuaauguagguuuc................	4	1	seq
......................................................................aagauguucCuaauguagguuuc................	13	1	seq
......................................................................aagauguucuAaauguagguuuc................	42	1	seq
......................................................................aagauguucuuaauCuagguuuc................	1	1	seq
......................................................................aagaugCucuuaauguagguuuc................	5	1	seq
```

```
                              Star                                              Mature
ugagcccucgcggucauaucacaagaaccuauauuugggacagcucucauggaacucuugauaucauagaaagauguucuuaauguagguuucuugugcugaccuuuau
......................................................................aagauguucuuaaCguagguuuc................    14      1      seq
......................................................................aagauguucuuaauguaggAuuc................    32      1      seq
......................................................................aagauCuucuuaauguagguuuc................    1       1      seq
......................................................................aagauguucuuaauguGgguuuc................    14      1      seq
......................................................................aagauguucuuaauguagguNuc................    3       1      seq
......................................................................Uagauguucuuaauguagguuuc................    4       1      seq
......................................................................aagauguucuuaauguaCguuuc................    2       1      seq
......................................................................aagauguucuuaauguaAguuuc................    26      1      seq
......................................................................aagauguuGuuaauguagguuuc................    7       1      seq
......................................................................aagauguucuuGauguagguuuc................    15      1      seq
......................................................................aagauguucuuaaugAagguuuc................    37      1      seq
......................................................................aagauguucuCaauguagguuuc................    104     1      seq
......................................................................aagauguucuuaauguagAuuuc................    6       1      seq
......................................................................aagaGguucuuaauguagguuuc................    7       1      seq
......................................................................aagauguuUuuaauguagguuuc................    16      1      seq
......................................................................aagauguucuuaGuguagguuuc................    11      1      seq
......................................................................aagauUuucuuaauguagguuuc................    5       1      seq
......................................................................aagauguucuGaauguagguuuc................    5       1      seq
......................................................................aagauguuAuuaauguagguuuc................    20      1      seq
......................................................................aagaAguucuuaauguagguuuc................    34      1      seq
......................................................................Gagauguucuuaauguagguuuc................    20      1      seq
......................................................................aagauguucuuaauguaggCuuc................    716     1      seq
......................................................................aagauguucuuaaugCagguuuc................    8       1      seq
......................................................................aUgauguucuuaauguagguuuc................    9       1      seq
......................................................................aagauguucuuaauguagguuAc................    15      1      seq
......................................................................aagauguucuuaauguagguCuc................    89      1      seq
......................................................................Cagauguucuuaauguagguuuc................    2       1      seq
......................................................................aagauguucuuaaugGagguuuc................    4       1      seq
......................................................................aagauguucuuaaAguagguuuc................    17      1      seq
......................................................................aagauguucuuaauguagguAuc................    27      1      seq
......................................................................aagauguAcuuaauguagguuuc................    23      1      seq
......................................................................aagauguucuuaauguagguuuU................    254     1      seq
......................................................................aagaCguucuuaauguagguuuc................    10      1      seq
......................................................................aagauguucNuaauguagguuuc................    1       1      seq
......................................................................aagauguucuuaauguaAguuucu...............    1       1      seq
......................................................................aagauguucuuaauguaggCuucu...............    3       1      seq
......................................................................aNgauguucuuaauguagguuucu...............    1       1      seq
......................................................................aagauguucuuaaAguagguuucu...............    1       1      seq
......................................................................Nagauguucuuaauguagguuucu...............    1       1      seq
......................................................................aagauguucuuaauguGgguuucu...............    1       1      seq
......................................................................aagauguucuuaauguagguuCcu...............    1       1      seq
......................................................................aagauguucuuaauguagguuucu...............    6500    0      seq
......................................................................aagauguCcuuaauguagguuucu...............    1       1      seq
......................................................................aagauguucuuaauguagguuuAu...............    7       1      seq
......................................................................aagauguucuuaauguagguuGcu...............    1       1      seq
......................................................................aagauguucuuaaugCagguuucu...............    3       1      seq
......................................................................aagauAuucuuaauguagguuucu...............    1       1      seq
......................................................................aagaugAucuuaauguagguuucu...............    5       1      seq
......................................................................aagauguucuuaauguUgguuucu...............    1       1      seq
......................................................................aagauguucuAaauguagguuucu...............    3       1      seq
......................................................................aagauguucuuaauguagguAucu...............    5       1      seq
......................................................................aagauguucuuaUuguagguuucu...............    1       1      seq
......................................................................aUgauguucuuaauguagguuucu...............    1       1      seq
......................................................................aaUauguucuuaauguagguuucu...............    1       1      seq
......................................................................aagauguucuuaaugAagguuucu...............    1       1      seq
......................................................................aagaAguucuuaauguagguuucu...............    1       1      seq
......................................................................aagauguucAuaauguagguuucu...............    2       1      seq
......................................................................aagaCguucuuaauguagguuucu...............    1       1      seq
......................................................................aagauguucuuaauguaggGuucu...............    1       1      seq
......................................................................aagauguAcuuaauguagguuucu...............    4       1      seq
......................................................................aagauguucuuaauUuagguuucu...............    1       1      seq
......................................................................aagauguucuuaauguagguuuUu...............    23      1      seq
......................................................................aagauguucuuaauguaggAuucu...............    2       1      seq
......................................................................aagauguuAuuaauguagguuucu...............    3       1      seq
......................................................................aagauguucuuaaCguagguuucu...............    5       1      seq
......................................................................aagauguucuuaauguagguuAcu...............    1       1      seq
......................................................................aagauguucuuaauguagguuucA...............    350     1      seq
......................................................................aagauguucuuGauguagguuucu...............    2       1      seq
......................................................................aagauguucuuaauguagguuucC...............    2       1      seq
......................................................................aagaugCucuuaauguagguuucu...............    3       1      seq
```

```
                          Star                                        Mature
ugagcccucgcggucauaucacaagaaccuauauuugggacagcucucauggaacucuugauaucauagaaagauguucuuaauguagguuucuugugcugaccuuuau
......................................................................aagauguucuuaauguagguuucG...............   8    1    seq
......................................................................Gagauguucuuaauguagguuucu...............   1    1    seq
......................................................................aagauguucuuaauguagguuucuA..............   2    1    seq
......................................................................aagauguucuuaauguagguuucAu..............   19   1    seq
......................................................................aagauguucuuaauguagguuucuu..............   29   0    seq
......................................................................aagauguucuuaauguagguuuUuu..............   5    1    seq
......................................................................aagauguucuuaauguagguuucuG..............   1    1    seq
......................................................................aagauguucuuaauguagguuucuuU.............   4    1    seq
.......................................................................agauguucuuaauguagguu..................   6    0    seq
.......................................................................agauguucuuaauguagguuuc................   4    0    seq
.......................................................................agauguucuuaauguaggCuuc................   1    1    seq
.......................................................................agauguucuuaauguagguuucu...............   1    0    seq
........................................................................gauguucuuaauguagguu..................   10   0    seq
........................................................................gauguucuuaauguagguuu.................   3    0    seq
........................................................................gauguucuuaauguagguuucA...............   2    1    seq
.........................................................................auguucuuaauguagguu..................   3    0    seq
..........................................................................uguucuuaauguagguuu.................   16   0    seq
..........................................................................Aguucuuaauguagguuuc................   1    1    seq
..........................................................................uguucuuaauguagguuuc................   107  0    seq
..........................................................................uguucuuaauguagguuucu...............   1    0    seq
...........................................................................guucuuaauguagguuuc................   1    0    seq
```
